# Rac Activation by the T-Cell Receptor Inhibits T Cell Migration

**DOI:** 10.1371/journal.pone.0012393

**Published:** 2010-08-25

**Authors:** Eva Cernuda-Morollón, Jaime Millán, Mark Shipman, Federica M. Marelli-Berg, Anne J. Ridley

**Affiliations:** 1 Histocompatibility and Transplantation Unit, Hospital Universitario Central de Asturias, Oviedo, Spain; 2 Department of Cell Biology and Immunology, Centro de Biología Molecular Severo Ochoa, Cantoblanco, Spain; 3 Ludwig Institute for Cancer Research, University of Oxford, Oxford, United Kingdom; 4 Department of Immunology, Imperial College London, London, United Kingdom; 5 Randall Division of Cell and Molecular Biophysics, King's College London, London, United Kingdom; CNRS UMR6543, Université de Nice, Sophia Antipolis, France

## Abstract

**Background:**

T cell migration is essential for immune responses and inflammation. Activation of the T-cell receptor (TCR) triggers a migration stop signal to facilitate interaction with antigen-presenting cells and cell retention at inflammatory sites, but the mechanisms responsible for this effect are not known.

**Methodology/Principal Findings:**

Migrating T cells are polarized with a lamellipodium at the front and uropod at the rear. Here we show that transient TCR activation induces prolonged inhibition of T-cell migration. TCR pre-activation leads to cells with multiple lamellipodia and lacking a uropod even after removal of the TCR signal. A similar phenotype is induced by expression of constitutively active Rac1, and TCR signaling activates Rac1. TCR signaling acts via Rac to reduce phosphorylation of ezrin/radixin/moesin proteins, which are required for uropod formation, and to increase stathmin phosphorylation, which regulates microtubule stability. T cell polarity and migration is partially restored by inhibiting Rac or by expressing constitutively active moesin.

**Conclusions/Significance:**

We propose that transient TCR signaling induces sustained inhibition of T cell migration via Rac1, increased stathmin phosphorylation and reduced ERM phosphorylation which act together to inhibit T-cell migratory polarity.

## Introduction

T cells play a pivotal role in the immune response against infections, in transplant rejection, and autoimmune diseases. T cell migration is essential for their recruitment to sites of inflammation, but only T cells specific for relevant antigens are retained at these sites [1], implying that their migration is selectively inhibited by interaction with antigen (Ag) presenting cells (APCs). This activation involves the recognition of a specific Ag presented by major histocompatibility complex molecules to the TCR, together with co-stimulatory signals that cooperate to activate the T cell fully. T cell activation initiates a cascade of signals leading to cell proliferation and cytokine production. TCR engagement also rapidly induces a stop signal to inhibit T cell migration, allowing stable conjugate formation between T cells and APCs [2]–[4]. However, although signalling by the TCR has been extensively studied, little is known of the mechanisms that stop migration [4].

In order to migrate, T cells undergo a dramatic re-organization of membrane domains and the cytoskeleton to acquire a polarized morphology with an actin-rich lamellipodium at the front and a uropod at the rear [4]–[6]. The formation of these structures is controlled by members of the Rho GTPase family of proteins, including RhoA, Rac1/2 and Cdc42 [6], [7]. These GTPases cycle between an active GTP-bound form and inactive GDP-bound form, and regulate a variety of cytoskeletal molecules [8], [9]. In migrating cells, Rac and Cdc42 proteins are believed to stimulate actin polymerization at the leading edge to induce extension of lamellipodia and filopodia respectively while RhoA regulates actomyosin contractility and retraction of the rear of the cell [10].

Lymphocytes express the closely related isoforms Rac1 and Rac2. T cells derived from Rac2-deficient mice have defects in TCR-induced signaling and proliferation [11] as well as reduced chemotaxis to chemokines [12]. T cells deficient for Rac1 and Rac2 appear to be more strongly defective in TCR-stimulated signaling than cells lacking only Rac1 or Rac2, suggesting some functional redundancy between the two isoforms [13]. So far the consequence of Rac1 knock-out on T cell chemotaxis has not been addressed, but expression of dominant negative Rac1 inhibits T cell migration [14]. RhoA is also implicated in T cell polarization [14], uropod protrusion [15] and uropod retraction [16]. Rho GTPases affect cell polarity in part by regulating the localization and activity of the ezrin/radixin/moesin (ERM) family of proteins [7]. ERM proteins bind to a variety of membrane receptors and to phosphoinositides through their N-terminal FERM domain, and to actin filaments via their C-terminus, thereby acting as linkers between the actin cytoskeleton and membrane receptors. ERM proteins are important for uropod formation in T cells, and several ERM-binding receptors, including CD44 and ICAM-3, co-localize with ERM proteins in the uropod [7], [15]. Their activity is stimulated by C-terminal threonine phosphorylation (T558 in moesin) [17], which unfolds the proteins from an autoinhibited conformation, and is mediated by a number of different kinases depending on cell type and cell cycle status [7], [18].

Here we report that activation of the TCR inhibits T cell polarization and migration even after the TCR stimulus is removed, and that this involves increased Rac activity, which induces decreased ERM phosphorylation, increased stathmin phosphorylation and loss of a stable uropod together with formation of multiple lamellipodial protrusions. Expression of constitutively active Rac1 similarly inhibits cell polarization, and this can be rescued by expression of phosphomimetic moesin-T558D. Increased Rac activity following TCR engagement therefore leads to loss of a stable migratory polarity by co-ordinately increasing frontness signals (lamellipodia) and decreasing backness signals (uropod).

## Results

### TCR activation inhibits T cell migration after removal of TCR stimulus

**Figure 1:**
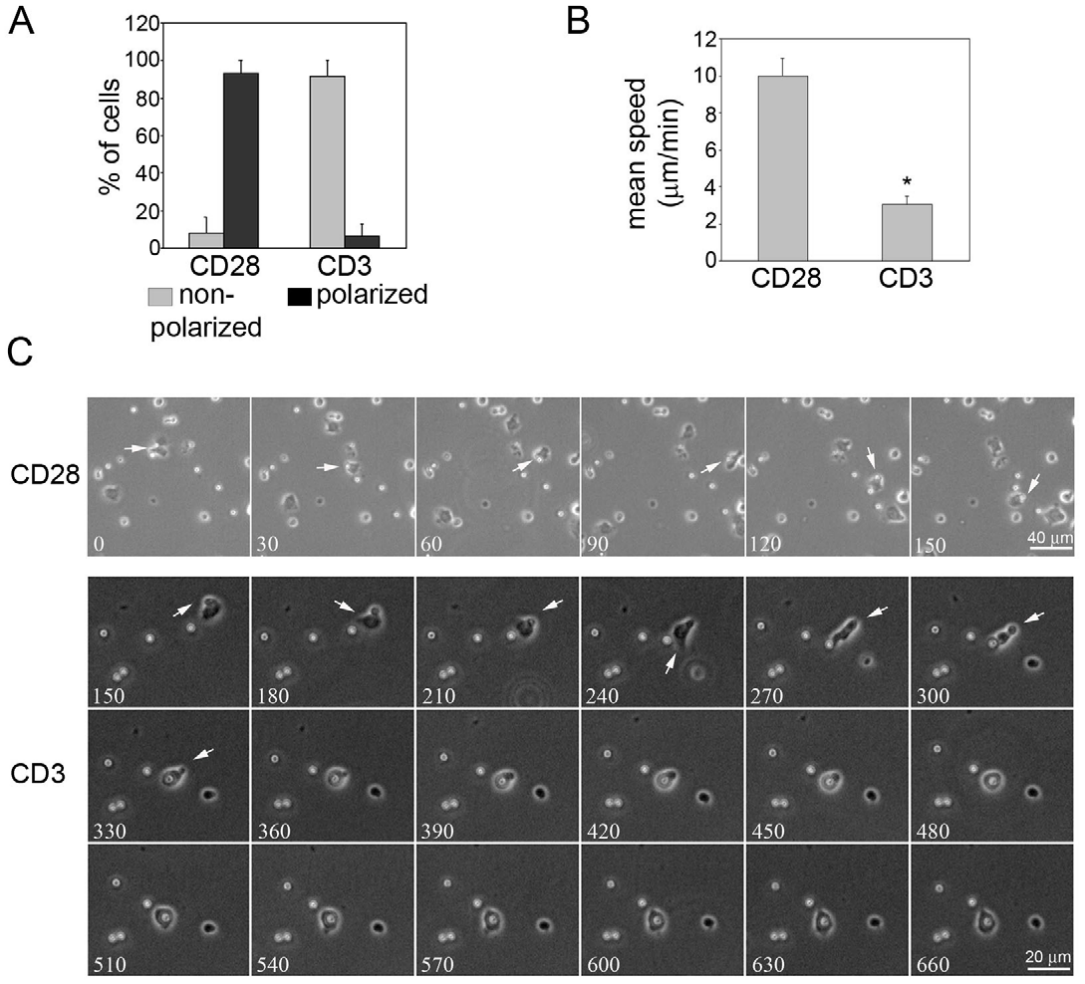
TCR activation by anti-CD3-coated beads impairs T cell polarization and migration. Beads coated with anti-CD3 or anti-CD28 (control) antibody were added to T cells migrating on ICAM-1 and images acquired every 30 sec. (A) Cells displaying a non-polarized (grey bars) or polarized (black bars) morphology upon T cell-bead contact were counted. Mean of 3 independent experiments +/− S.E.M. (B) Cells were tracked upon bead-contact. The graph shows the mean migration speed +/− S.E.M. of 3 independent experiments, n = 7–10 cells/experiment. *p<0.05, Student's t-test. (C) Consecutive frames of time-lapse microscopy movies are shown. Numbers on each frame indicate seconds after the start of the movie. Arrows indicate a cell interacting with an antibody-coated bead in each condition. Scale bars, 40 µm (CD28) or 20 µm (CD3).

It has previously been reported that engagement of the TCR inhibits T-cell migration. Consistent with this, when T cells migrating on ICAM-1-coated surfaces encountered anti-CD3 antibody (OKT3)-coated beads we observed rapid inhibition of cell polarization and migration upon TCR engagement. In contrast, control anti-CD28 antibody-coated beads did not affect T-cell migration (Figure 1A–C, Movies S1, S2).

**Figure 2:**
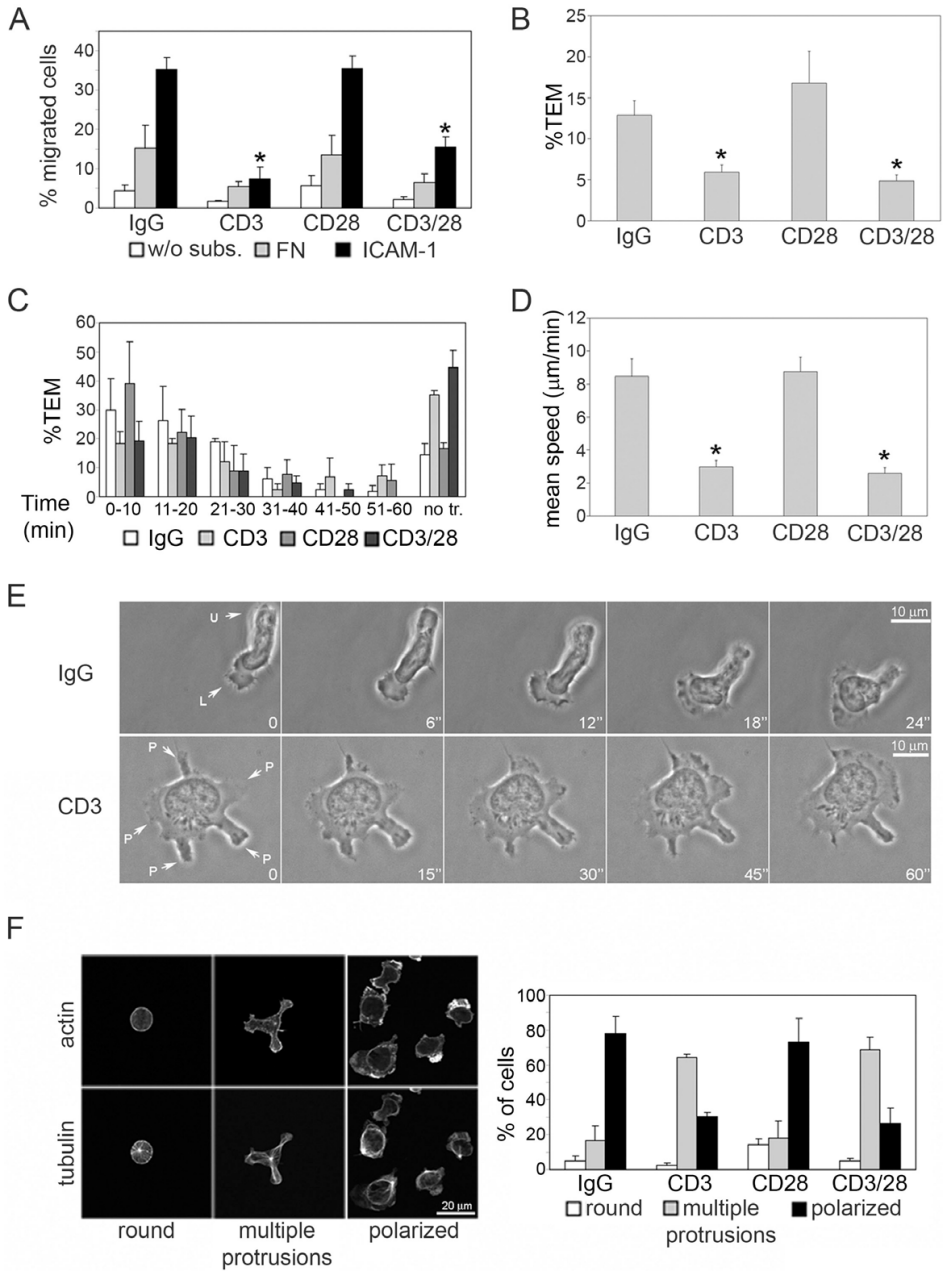
TCR activation inhibits T cell migration and impairs polarity. T cells were plated on the indicated antibodies for 45 min then removed from plates prior to each experiment. (A) Antibody-pre-stimulated T cells were added to transwell filters coated with fibronectin (FN; grey bars), ICAM-1 (black bars) or uncoated, without substrate (w/o subs; white bars). (B) Quantification of T cell TEM through TNF-α-activated endothelial cell monolayers on transwells. The number of migrated cells in the lower chamber was determined after 3 h. (C) TEM across endothelial cells on glass coverslips was analysed by time-lapse microscopy. The number of T cells that transmigrated within each 10-min interval from 0 to 60 min was determined; no tr.: cells that did not undergo transendothelial migration. Results (A–C) are the mean of 3 independent experiments +/− S.E.M. *p<0.05 compared to IgG, Student's t-test. (D,E) Antibody-pre-treated T cells were plated on ICAM-1 and imaged by time-lapse microscopy. (D) Migration speed of cells expressed as the mean of the average speed in five different experiments +/− S.E.M.; 50 cells per condition; * p<0.05 compared to IgG, Student's t-test. Scale bar, 10 µm. (E) Representative images of a control IgG-pre-stimulated and CD3-pre-stimulated T cell on ICAM-1. L: lamellipodium/leading edge, U: uropod, P: protrusion. Numbers on each frame indicate seconds after the start of the movie. Scale bar  = 10 µm. (F) Antibody-treated cells were plated on ICAM-1 for 30 min, then fixed and stained for F-actin and tubulin. Morphologies were classified into three groups based on F-actin distribution and MTOC localization (left panels; Scale bar, 20 µm). Mean of % of cells displaying each morphology in 5 independent experiments +/− S.E.M. (right panel, 100 cells per condition in each experiment).

To determine whether the effect of TCR stimulation on migration is maintained after removal of the TCR stimulus, TCR signaling was activated by plating T cells on anti-CD3 antibodies for 45 min, a length of time similar to the formation of a mature immunological synapse [19]. Cells were subsequently removed from the plates and added to transwell filters coated with ICAM-1 or fibronectin, which are ligands for the T-cell integrins LFA-1 (αLß2) and/or VLA-4 (α4ß1) respectively (Figure 2A). We found that TCR stimulation for 45 min was sufficient to inhibit T-cell migration on ICAM-1 in the absence of ongoing TCR engagement. Similar results were obtained when T cells were pre-incubated with different antibodies to the TCR (Figure S1A). The T cells used in our assays are activated by stimulation with phytohemagglutinin (PHA) and IL-2, and resemble memory T cells [20]. Co-incubation of cells with anti-CD28 and anti-CD3 antibodies did not alter the inhibitory effect of CD3 on migration (Figure 2A), consistent with the observation that memory T cells and human T cells activated in vitro are not dependent on CD28 co-stimulation for TCR-induced responses [21], [22]. Although it has been reported that T cell migration is increased upon CD28 cross-linking [20], under our experimental conditions CD28 activation with plate-bound antibody did not affect subsequent migration.

TCR engagement transiently activates LFA-1 and increases the strength of adhesion to ICAM-1, but this is reversed by 30 min [23]. Consistent with this, under our conditions (45 min of CD3 engagement), subsequent adhesion of T cells to ICAM-1 following removal from anti-CD3 antibodies was not altered (Figure S1B), indicating that the effect of CD3 activation on T cell migration was not due to changes in cell adhesion. T cell transendothelial migration (TEM) requires adhesion to endothelial ICAM-1. As well as reducing migration of CD3-activated cells on ICAM-1-coated surfaces, pre-treatment of T cells with anti-CD3 antibody efficiently inhibited TEM and delayed the time at which TEM occurred (Figure 2B and C). Co-incubation with anti-CD3 and anti-CD28 antibodies again did not significantly alter migration compared to CD3 alone. CD3 activation therefore inhibits T cell migration without affecting cell adhesion.

### TCR activation inhibits T cell polarization on ICAM-1

To identify the stage at which TCR engagement affects T cell migration, we monitored the migration of CD3 antibody pre-treated cells on ICAM-1 by time-lapse microscopy. Most control T cells had a migratory phenotype, characterized by a clear polarized morphology (Figure 2E, F, Movie S3), with a single lamella containing an F-actin-rich lamellipodium and highly dynamic membrane ruffles, and a uropod frequently protruding out of the plane of migration. Migrating T cells sometimes extended multiple lamellae but one quickly became dominant and formed the leading edge. By contrast, following CD3 stimulation, the majority of cells were unpolarized with multiple actively ruffling protrusions enriched in F-actin and no well-defined uropod (Figure 2E, F, Movie S4). These protrusions were relatively stable compared to the occasional transient multiple lamellae observed in control cells, but eventually collapsed into the body of the cell as new protrusions formed in other regions. (Figure S1C, Movie S4). Consistent with the absence of migratory polarity, CD3 pre-treatment also reduced cell migration speed on ICAM-1 and the total distance moved (Figure 2D, Figure S1D).

We then analysed whether the CD3-induced phenotype correlated with changes in the cytoskeleton. Control T cells migrating on ICAM-1 had an F-actin-rich lamellipodium, whereas microtubules were concentrated in the uropod and the microtubule-organizing centre (MTOC) was behind the nucleus, as previously observed [5]. In contrast, CD3-pre-activated cells had no preferential actin filament accumulation in one part of the cell, lacked a well-defined uropod and the MTOC was generally positioned centrally (Figure 2F). The loss of polarity was confirmed by staining for several membrane proteins that are normally localized in the uropod of polarized cells, including CD44 and ICAM-3 (Figure S1E) [6]. These markers displayed a homogeneous distribution at the cell surface in cells pre-treated with anti-CD3 antibodies.

### Effect of TCR activation on Rho GTPase activity and localization

**Figure 3:**
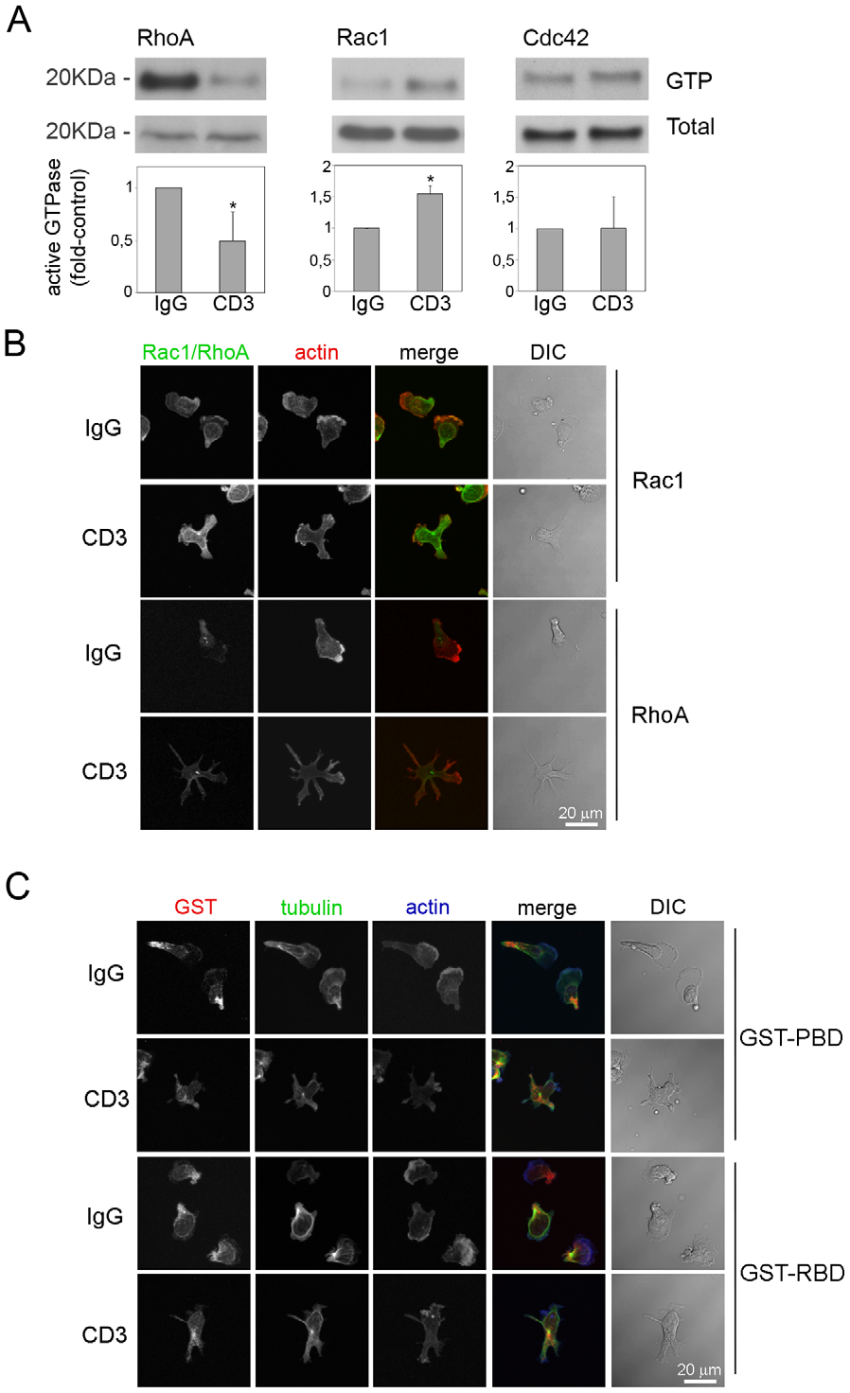
Regulation of Rho GTPase activity and localization by TCR activation. (A) T cells were starved for 5 h and incubated in anti-CD3 or control IgG antibody-coated wells. After 45 min cells were lysed and RhoA, Rac1 and Cdc42 activity assayed in pull-down experiments. A representative experiment out of at least 3 for each Rho GTPase is shown. Graphs show active Rho GTPase (GTP) levels:total levels as mean of 5 experiments (RhoA and Rac1) or 3 experiments (Cdc42) +/− S.E.M. relative to IgG pre-treated cells. *p<0.05, Students t-test. (B,C) Localization of total Rac1 and RhoA (B) and active Rac and Rho GTPases (C) in cells plated on ICAM-1. To detect active Rac and Rho, cells were incubated with purified GST-PAK-PBD (GST-PBD) and GST-Rhotekin-RBD (GST-RBD) respectively followed by anti-GST antibodies. Cells were also incubated with fluorophore-conjugated phalloidin to localize F-actin and anti-tubulin antibodies as indicated. DIC: differential interference contrast image. Scale bars, 20 µm.

Since Rho GTPases are known to play a pivotal role in regulating actin and microtubule dynamics, the effect of TCR signalling on RhoA, Rac1 and Cdc42 was explored. CD3 stimulated Rac1 activation in T cells, consistent with previous observations in the Jurkat T cell line [24], [25], and decreased RhoA activity, whereas no change in Cdc42 activity was observed (Figure 3A).

A balance between Rac and RhoA activities has been proposed to be necessary for the process of neutrophil polarization, with Rac activation promoting the leading edge “frontness” and locally inhibiting RhoA, and RhoA activity at the rear of the cells determining the “backness” [26]. The localization of both total and active forms of Rac and RhoA was altered in CD3 pre-stimulated cells (Figure 3B, C). In control cells, Rac1 localized in lamellipodia and membrane ruffles, around the nucleus and in the uropod, whereas in CD3-pre-activated cells it had a more homogeneous distribution (Figure 3B). The localization of active Rac in cells was determined by incubating fixed cells with purified GST-PAK-PBD (binds to Rac-GTP). Control staining of cells expressing constitutively active Rac1, Cdc42 (Figure S2C) or dominant negative Rac1 (data not shown) indicated that GST-PAK-PBD recognized the active form of Rac1 in cells but not Cdc42. Active Rac localized to the membrane at the leading edge (Figure 3C), as well as in the uropod, where it was predominantly in vesicular structures around the MTOC and not on the plasma membrane. This localization of active Rac is similar to results in neutrophils, where it is found in lamellipodia and the uropod [27]. In anti-CD3 pre-treated cells active Rac was enriched in many of the multiple protrusions (Figure 3C), consistent with a role in promoting lamellipodium extension. Both total RhoA and active Rho (note that the GST-Rtk-RBD probe does not differentiate between RhoA, RhoB and RhoC) normally localized predominantly to the uropod with some localization at the front in lamellipodia, but was found in all protrusions in CD3-pre-activated cells (Figure 3B, C). Active Rho was also found close to but did not co-localize with the MTOC in CD3-pre-treated cells.

### TCR activation decreases phosphorylation of ERM proteins and alters their localization

**Figure 4:**
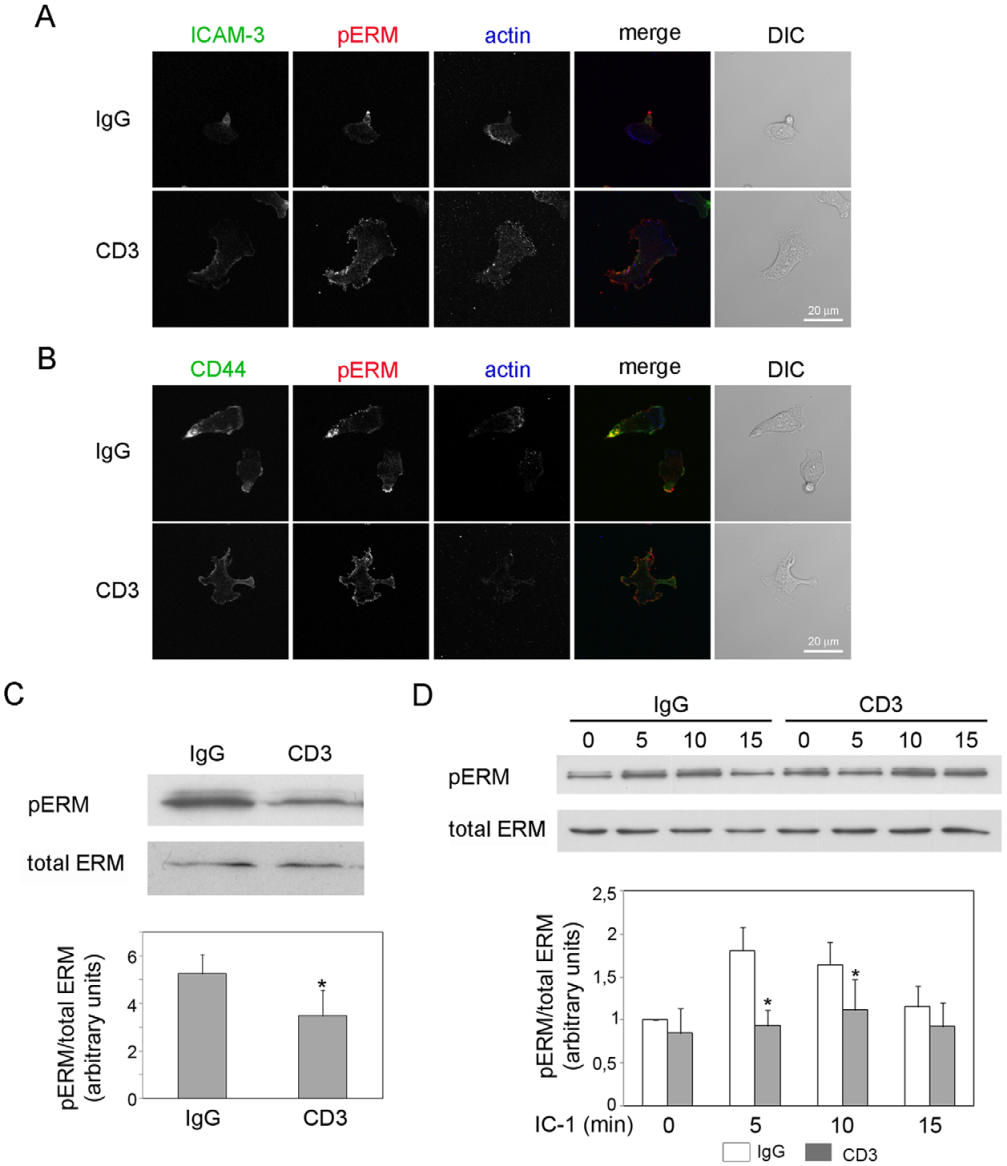
TCR activation promotes ERM dephosphorylation and inhibits uropod localization of pERMs. (A–D) T cells were plated on anti-CD3 antibody or control IgG for 45 min. (A, B) Antibody-pre-treated cells were plated on ICAM-1. After 30 min cells were fixed with TCA and the localization of pERM and actin (A, B), ICAM-3 (A) and CD44 (B) assessed by immunofluorescence. Representative confocal images are shown. DIC: differential interference contrast image. Scale bars, 20 µm. (C) Cells were lysed and the levels of phosphorylated ERM proteins (pERM) determined by western blotting. A representative blot and the mean values of 3 independent experiments +/− S.E.M. are shown. *p<0.05, Students t-test. (D) Antibody-pre-treated cells were plated on ICAM-l or kept in suspension (t = 0), lysed at the indicated time-points and the levels of pERM determined by western blotting. A representative blot and the mean values of 4 independent experiments +/− S.E.M. compared to IgG-pre-treated cells at t = 0 are shown. *p<0.05, Students t-test compared to IgG pre-treated cells at the same time point.

The uropod is believed to be important for T cell migration, allowing cells to migrate through constricted spaces and also participating in the recruitment of other cells [28]. C-terminal phosphorylation and activation of ERM proteins is necessary for posterior localization of ERM-binding receptors such as ICAM-3 and CD44 [29], and cooperates with the Rho/ROCK pathway in stimulating uropod projection [15]. On the other hand, TCR engagement induces dephosphorylation of ERM proteins via Rac in T cells, which is proposed to enhance T cell interaction with APCs [30]. Since anti-CD3-pre-treated cells lacked uropods when plated on ICAM-1, we investigated the localization of phosphorylated ERM proteins. In control cells, phosphorylated ERM proteins (pERM) and the uropod markers ICAM-3 and CD44 were localized primarily in the uropod as expected, whereas in anti-CD3-pre-treated cells they localized to multiple patches on the plasma membrane (Figure 4A, B). We then analyzed the phosphorylation status of ERM proteins following CD3 activation. ERM phosphorylation was decreased by CD3 engagement (Figure 4C), but this was reduced following detachment of cells from anti-CD3 antibody (Figure 4D, t = 0). However, CD3 pre-activation did inhibit the transient increase in ERM phosphorylation induced by adhesion to ICAM-1 (Figure 4D). Since ERM phosphorylation is required for uropod formation, this reduction in ERM phosphorylation on ICAM-1 could be responsible for the loss of uropods in anti-CD3-pre-treated cells. These results indicate that transient TCR engagement induces a prolonged inhibition of polarization of uropod proteins and formation of a uropod.

### TCR activation increases stathmin phosphorylation and changes MT distribution

**Figure 5:**
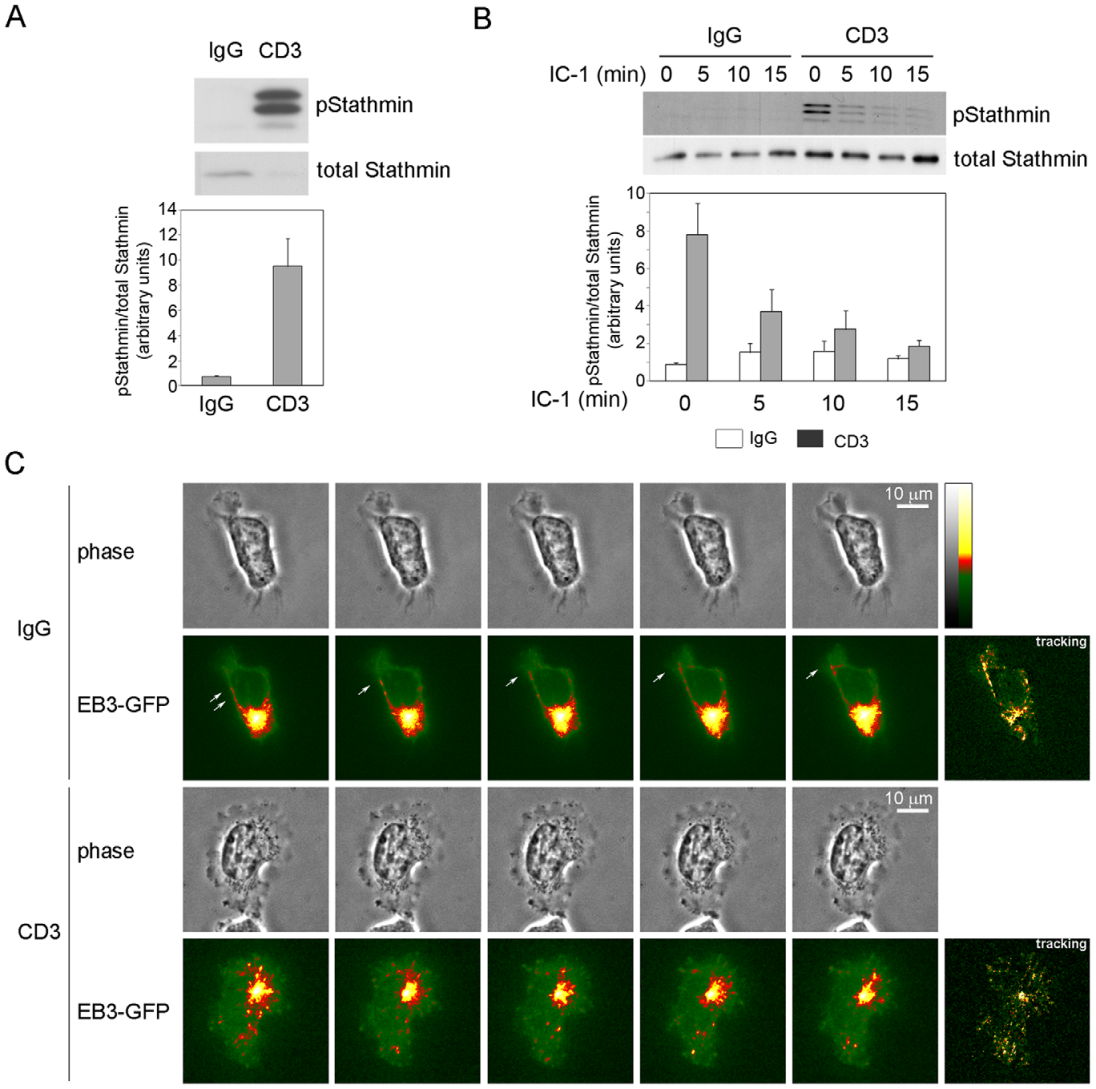
TCR activation increases stathmin phosphorylation and alters microtubule organization. T cells were plated on anti-CD3 antibody or control IgG for 45 min. (A) Cells were lysed and the levels of phospho-stathmin (pStathmin) and total stathmin assessed by western blotting. The graph shows relative levels of phospho-stathmin (normalized to total stathmin) of 3 independent experiments +/− S.E.M. in arbitrary units. (B) Antibody pre-treated T cells were collected, plated on ICAM-1 (IC-1) and lysed at the indicated time points. The levels of phospho-stathmin and total stathmin were assessed by western blotting. Representative blots are shown; the graph shows mean levels of phospho-stathmin (normalized to total stathmin) of 3 independent experiments +/− S.E.M. relative to IgG-treated cells at t = 0. (C) Antibody pre-treated cells expressing EB3-GFP were plated on ICAM-1-coated coverslips. Images were collected every 5 sec. Images shown correspond to five consecutive frames, from 130 sec after the start of the movie. Greyscale images (256 greyscale) were converted to indexed color (scale on right) to improve the visualization of small differences in intensity value. Arrows indicate individual microtubule tips moving towards the leading edge. Tracking images (right panels) show the movement of EB3-GFP in the 5 frames (see Methods for detail). Scale bars, 10 µm.

**Figure 6:**
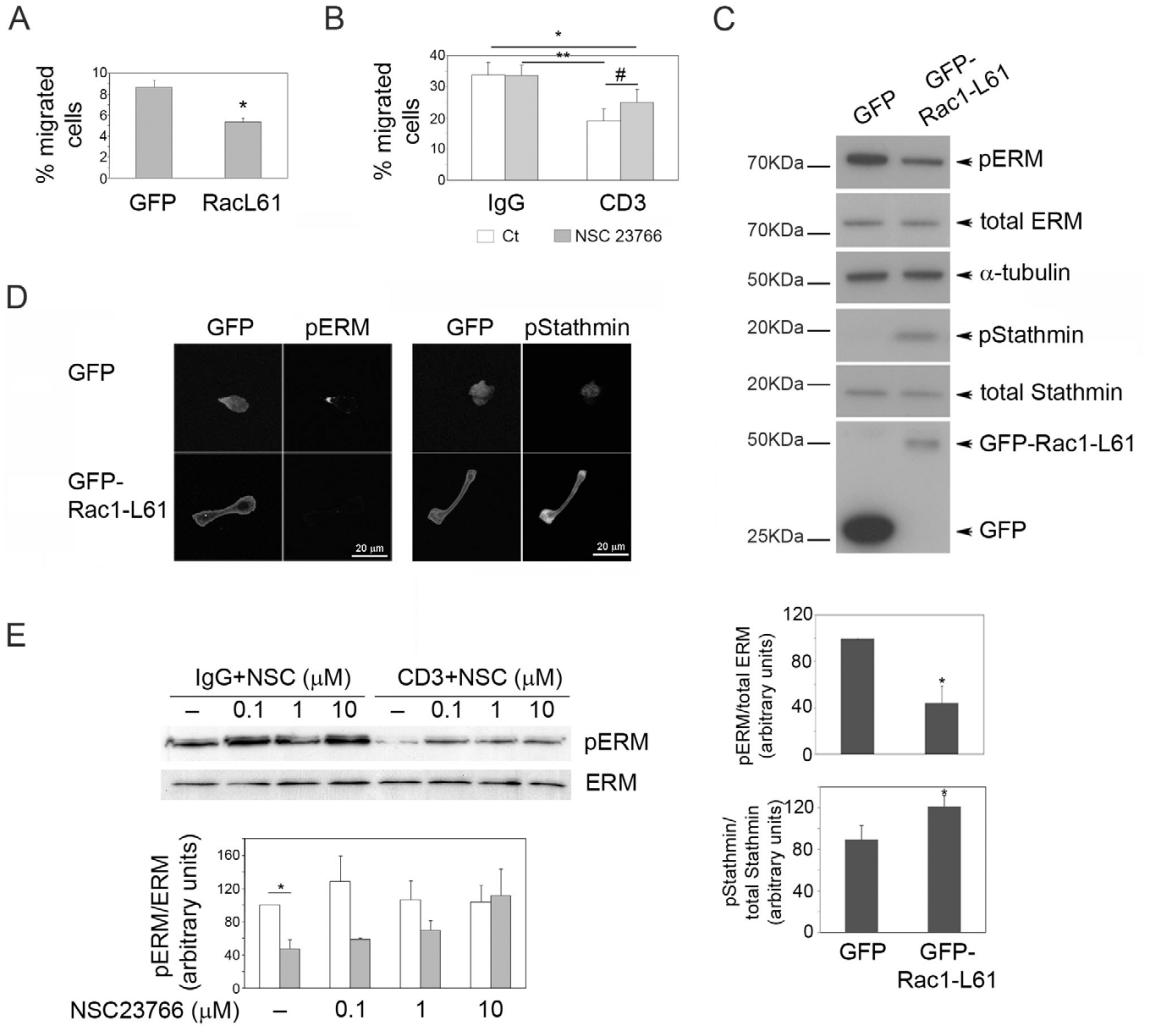
Rac activation is required for TCR-induced inhibition of migration and polarity. (A) T cells expressing GFP or GFP-Rac1-L61 (constitutively active) were assayed for migration in ICAM-1-coated transwells. The mean of 3 independent experiments +/− S.E.M. is shown; *p<0.01 compared to GFP, Student's t-test. (B) T cells treated with anti-CD3 antibodies or IgG, in the absence (open bars) or presence (grey bars) of the Rac inhibitor NSC23766, were collected in starving medium, washed and assayed for migration in ICAM-1-coated transwells. The mean of 6 independent experiments +/− S.E.M. is shown; *p<0.05 compared to IgG, **p<0.05 compared to IgG+NSC23766, #p<0.005 compared to CD3, Student's t-test. (C) T cells expressing GFP or GFP-Rac1-L61 were lysed and the levels of the indicated proteins analyzed by western blotting. Representative blots are shown; the graphs show mean levels of pERM/ERM and pStathmin/total stathmin for 3 experiments +/− S.E.M. in GFP-Rac1-L61-transfected cells relative to GFP-transfected cells *p<0.05, Students t-test. (D) GFP-Rac1-L61 induces dephosphorylation of ERM and phosphorylation of stathmin. T cells expressing GFP-Rac1-L61 or GFP as control and adhered to ICAM-1 for 1 h were fixed and stained with antibodies to the indicated proteins. Scale bars, 20 µm. (E) Inhibition of Rac reverts CD3-induced ERM dephosphorylation. NSC23766-treated T cells were incubated with IgG (open bars) or anti-CD3 (grey bars) for 45 min. Expression of the indicated proteins was analysed by western blotting. A representative blot is shown; the graph shows mean levels of pERM/ERM for 3 experiments +/− S.E.M. *p<0.05, Students t-test.

As well as regulating the actin cytoskeleton and pERM, Rac1 has been proposed to stabilize microtubules in epithelial cells by PAK-induced phosphorylation and inactivation of stathmin, a microtubule-destabilizing protein [31]. Since microtubules can regulate T-cell polarity and migration [32] we analyzed the phosphorylation status of stathmin. We found that treatment of cells for 45 min with anti-CD3 antibody or transfection with constitutively active GFP-Rac1-L61 strongly increased stathmin phosphorylation (Figure 5A, 6C, D), in accord with previous observations [31], [33]. When T cells were subsequently plated on ICAM-1 in the absence of CD3 antibodies, they retained increased stathmin phosphorylation above control cells for at least 10 min (Figure 5B). This suggests that microtubule dynamics are altered by TCR engagement, and that this effect is maintained after TCR engagement is stopped.

To monitor microtubule dynamics in living cells, T cells were transfected with EB3-GFP, a microtubule tip-binding protein previously used to study microtubule dynamics [34]. EB3-GFP accumulated predominantly at microtubule tips in the uropod in migrating cells and was also detected moving towards the leading edge, along the migration axis (Figure 5C, upper panels and Movie S5). In anti-CD3 pre-treated cells, EB3-GFP moved from the cell centre outwards to the cell periphery without any preferential directionality (Figure 5C, lower panels and Movie S6). Microtubule organization is therefore altered by TCR stimulation.

### Rac contributes to TCR-induced inhibition of T cell migration

We then explored whether the higher Rac activity induced upon TCR stimulation was responsible for its effects on T cell morphology and migration. T cells expressing constitutively active Rac1, GFP-Rac1-L61, were similar in morphology to CD3-activated cells: they had multiple lamellae, lacked a distinguishable uropod, did not polarize on ICAM-1 and had impaired migration (Figure 6A, D, Movie S7). To reduce Rac activity, T cells were incubated with anti-CD3 antibody in the presence of the Rac inhibitor NSC23766 [35], [36]. NSC23766 (1 µM) partially rescued the CD3-induced inhibition of T cell migration through ICAM-1-coated transwells (Figure 6B). This concentration inhibited CD3-induced Rac1 activation but had little effect on RhoA activity (Figure S2B). Higher doses of NSC23766 strongly reduced basal Rac1 activity as well as RhoA activity, and inhibited T cell migration (data not shown), most likely because some Rac and RhoA activity is needed for migration [37]. It is therefore likely that TCR engagement activates Rac1 to a higher level than that required for migration, which then disrupts the balance between Rac1 and RhoA so that stable migratory polarity is lost.

T cells transfected with Rac1-L61 had decreased levels of pERM (Figure 6C), in agreement with the observation that dominant-negative Rac1 inhibits TCR-induced ERM dephosphorylation [30]. In addition, in Rac1-L61-expressing cells, pERM were uniformly distributed on the plasma membrane and no longer localized to a discrete uropod (Figure 6D), similar to pERM localization in CD3-activated cells (Figure 4A, B). Moreover, treatment of cells with NSC23766 increased pERM levels in CD3-activated cells (Figure 6E). The reduction in pERM following TCR engagement therefore involves Rac.

**Figure 7:**
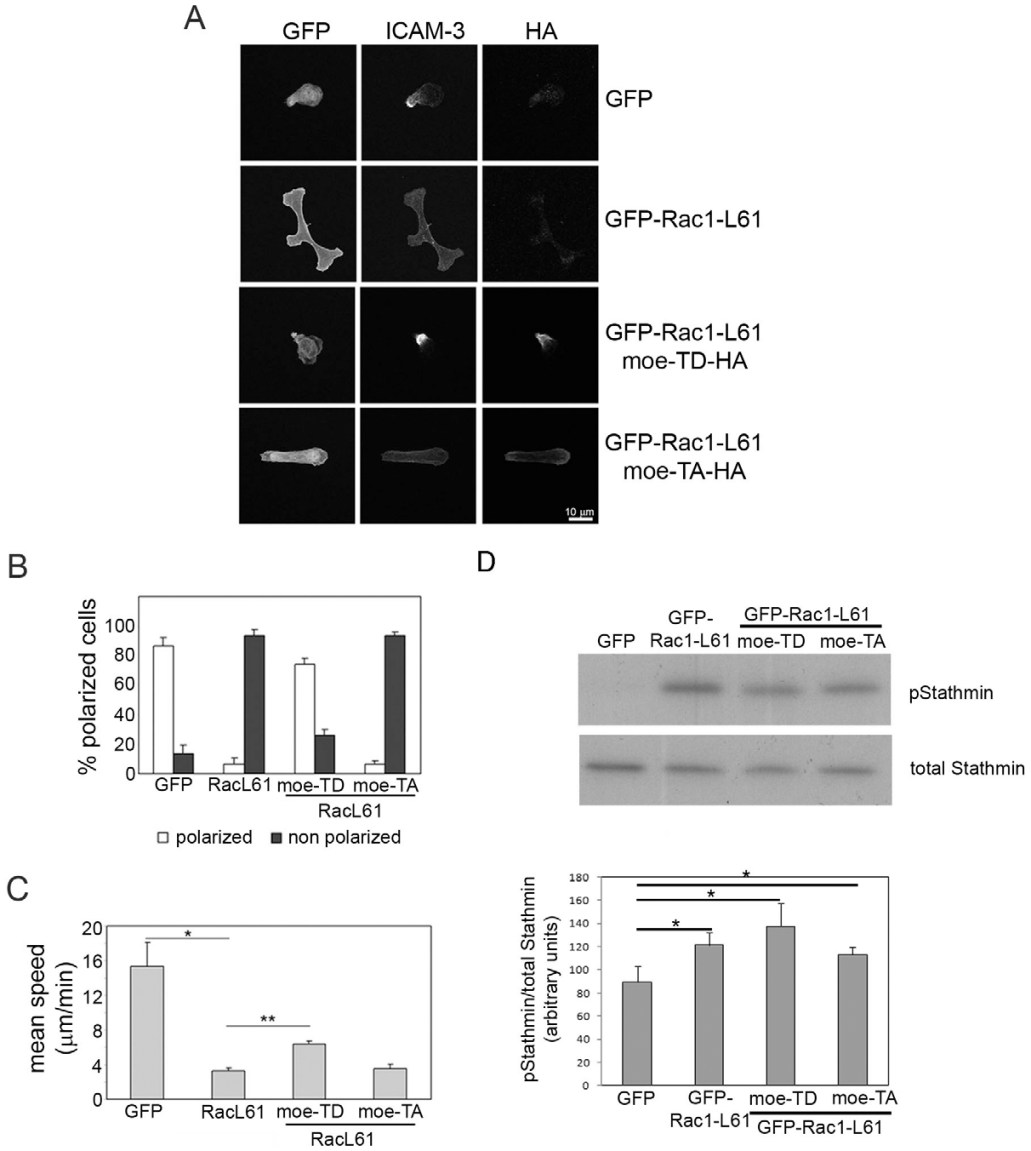
A phosphomimetic moesin mutant reverts Rac1-L61 inhibition of T cell polarization and motility. (A) Cells transfected with the indicated plasmids were stained for ICAM-3 and the HA tag on moesin. Moe-TD-HA, moesin-T558D-HA; Moe-TA-HA, moesin-T558A-HA. Representative images are shown. Scale bar, 10 µm. (B) Frequency of each cell morphology; mean of the % of cells in 3 independent experiments +/− S.E.M. Only transfected cells were analysed. Polarized: cells with uropod and clustering of ICAM-3 (open bars). Non-polarized: Cells with multiple protrusions or absence of uropod and homogeneously distributed ICAM-3 (black bars). (C) T cells expressing the indicated proteins were imaged by time-lapse microscopy on ICAM-1; migration speed of transfected cells pre-treated with the indicated antibodies is the mean of the average speed in four different experiments +/− S.E.M.; p<0.05, Student's t-test compared to GFP (*) or compared to GFP-Rac1-L61 (**). (D) T cells expressing the indicated proteins were lysed and the phosphorylation of stathmin analyzed by western blotting. A representative blot is shown; the graph shows mean levels of pStathmin/total stathmin for 3 experiments +/− S.E.M. *p<0.05, Student́s t-test.

To determine whether changes in ERM phosphorylation are important for TCR-induced inhibition of T cell polarization and migration, we investigated the effect of moesin mutants. The Rac1-L61-induced loss of polarized morphology and ICAM-3 localization to the uropod was rescued by co-expression of an activated form of moesin, moesin-T558D, which mimics C-terminal phosphorylation [38]. Moesin-TD-expressing cells had a clear uropod and polarized migratory phenotype (Figure 7A, B). In contrast, a non-phosphorylatable mutant (moesin-T558A) had no effect on ICAM-3 localization or cell polarization (Figure 7A, B). Moreover, moesin-TD but not moesin-TA partially reversed the Rac1-L61-induced decrease in T cell motility (Figure 7C). The ability of activated phospho-mimetic moesin-TD to rescue the polarity defect of cells expressing activated Rac1 indicates the importance of the uropod in establishing cell polarity. However, it was not able to rescue migration speed completely, probably because it did not revert the Rac1-induced increase in stathmin phosphorylation (Figure 7D) and hence microtubule dynamics.

## Discussion

**Figure 8:**
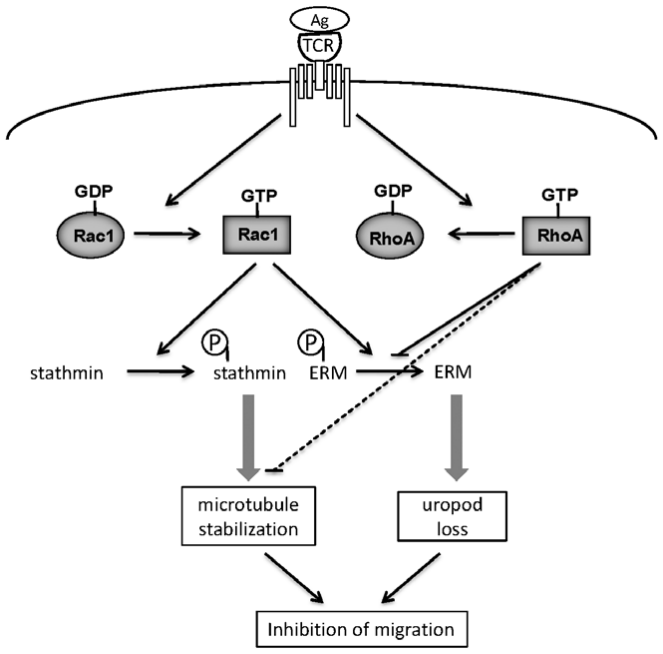
Schematic model for mechanisms leading to TCR-induced loss of migratory polarity. TCR engagement by antigen leads to activation of Rac1 (increased Rac1-GTP) and inhibition of RhoA (decreased RhoA-GTP). Active Rac1 stimulates stathmin phosphorylation, leading to microtubule stabilization, and reduces ERM phosphorylation, leading to loss of uropod structures. Active RhoA normally stimulates ERM phosphorylation and reduces microtubule stability, and these responses are reduced when RhoA activity decreases. Loss of the uropod together with increased microtubule stability reduces migratory polarity and hence inhibits migration.

TCR engagement activates many different signaling pathways that contribute to the formation of a stable T cell-APC interaction zone or ‘synapse’ and lead to T-cell activation, including proliferation and secretion of cytokines [4], [39]. TCR engagement is also known to induce a migration stop signal [4]. Here we have shown that the effect of TCR engagement in inhibiting migration is maintained even after removal of the TCR stimulus, and that cell migration is impaired for up to 2 hours. The signaling pathways responsible for the TCR stop signal have not previously been investigated in detail. Our results here indicate that TCR activation of Rac and subsequent decreased ERM phosphorylation and increased stathmin phosphorylation leads to prolonged loss of T-cell migratory polarity and hence inhibits migration (Figure 8). This would allow T cells to polarize their microtubules towards APCs and form a stable immunological synapse.

TCR engagement has previously been shown to induce transient ERM dephosphorylation via Rac, and this has been proposed to promote conjugate formation between T cells and APCs by reducing cortical rigidity [30], [40]. Our data indicate that this TCR-activated signaling pathway is also central to loss of migratory polarity in T cells, both inhibiting uropod formation and promoting extension of multiple protrusions. In our experiments with anti-CD3-coated beads, TCR engagement rapidly inhibits cell polarity and migration, consistent with previous observations of T cells migrating on ICAM-1-containing lipid bilayers with and without cognate antigen [3]. Interestingly, we find that the effect of TCR activation on signaling and migration does not require continuous stimulation, but is retained after removal of anti-CD3 antibody. We have observed that the inhibition of migration lasts up to two hours. This suggests that CD3 treatment induces stable changes in signaling to Rac, perhaps by decreasing the activities of proteins that normal turn off Rac activity, including GAPs or GDIs [9]. It would be interesting to know whether the TCR complex retains activity over this time. It is also possible that prolonged TCR signaling leads to the degradation of proteins that directly or indirectly regulate Rac; for example, RhoA has been shown to be targeted for degradation by the Smurf1 ubiquitin ligase [41]. This long-term inhibition of migration might be important for retaining T cells at a site of inflammation once they have encountered antigen, even if the APC subsequently dies, for example as the target of a cytotoxic T cell [42], or the TCR is endocytosed and downregulated [43].

Stathmin destabilizes microtubules, and phosphorylation of stathmin reduces its activity [44]. TCR stimulation and Rac1 strongly induce stathmin phosphorylation, which will stabilize microtubules. Microtubule stabilization has in turn been shown to activate Rac1 in fibroblasts [45]. We postulate that TCR-induced stathmin phosphorylation, which is sustained after removal of CD3 stimulation, could prevent T cell polarization on ICAM-1 by increasing Rac1 activity. In addition, we have shown that RhoA acts via ROCK to destabilize microtubules [32], and thus TCR-induced inhibition of RhoA could also contribute to changes in microtubule dynamics leading to loss of migratory polarity (Figure 8).

Rac1 and RhoA have opposing effects on ERM proteins in T lymphocytes: Rac stimulates ERM dephosphorylation while RhoA increases their phosphorylation [30], [46]. In migrating T cells the presence of active Rac at the leading edge would explain exclusion of phosphorylated ERM proteins from this region, while Rac activity detected at the rear of cells appears not to be associated with the plasma membrane but predominantly localized on vesicles, and is thus unlikely to affect ERM phosphorylation on the uropod membrane. On the other hand, RhoA is localized in the uropod of migrating T cells, and could therefore mediate the accumulation of phosphorylated ERM proteins in this region. Lymphocyte-oriented kinase (LOK) has recently been shown to regulate ERM phosphorylation in T cells and also affect migration [47], and thus it will be interesting to know if LOK acts in the uropod. In CD3-stimulated cells, increased Rac activation could be sufficient to account for the reduced ERM phosphorylation.

Altogether, our data support a model for prolonged TCR-induced inhibition of cell polarization and migration (Figure 8). A balance between Rac and RhoA activation is necessary for T cell migratory polarity, with Rac contributing to leading edge formation via actin polymerization, and RhoA together with phosphorylated ERM proteins to uropod formation. We propose that sustained Rac activation in CD3-activated cells prevents migratory polarity by inducing the formation of multiple protrusions, increasing microtubule stability and inducing loss of the uropod via decreased ERM phosphorylation. Together, these effects result in the loss of a polarized migratory morphology and prevent efficient migration, and could therefore account for T cell retention upon antigen recognition in sites of inflammation and infection.

## Materials and Methods

### Cell culture and receptor activation

T lymphocytes were prepared as previously described [48] and cultured in RPMI-1640 containing 10% human AB serum (BioWest) and 10 U/ml IL-2 (Roche). Experiments were performed after culturing cells for 10 to 15 days. Human umbilical vein endothelial cells (HUVECs; BioWhittaker) were cultured as previously described [48].

For receptor activation T cells were incubated for 5 h in starving medium (RPMI 1640 containing 1% heat-inactivated FCS). Wells were coated with monoclonal antibodies against CD3 (OKT3, 1 µg/ml; a gift from Dr. M. Garin, Imperial College London, UK, or purchased from BioLegend; or for Fig. S1A, MEM57 and MEM92, a gift from Dr. V Horejsi, Institute of Molecular Genetics, Prague, Czech Republic), CD28 (5 µg/ml; BD Bioscience) or control mouse IgG in 50 mM Tris-HCl, pH 9.5 (3 h, 37°C) and blocked with 2.5% BSA/PBS (1 h). For some experiments OKT3 was obtained from Cells were incubated in antibody-coated wells for 45 min and collected with starving medium. Alternatively, T cells were incubated with 6-µm Polybead Microspheres (PolyScience), which had been coated with anti-CD3 or anti-CD28 antibodies (16 h, 4°C) and blocked in 1% BSA/PBS.

### T cell migration and transendothelial migration

For transwell assays, T cells were incubated in starving medium for 5 h, incubated with plate-bound antibodies for 45 min then added to 3-µm pore transwell membranes coated with human fibronectin (2 µg/ml; Sigma-Aldrich), ICAM-1-Fc (5 µg/ml; R&D Systems) or uncoated. Where indicated, cells were incubated with the Rac inhibitor NSC23766 (Calbiochem). For transendothelial migration (TEM), starved T cells (3×10^5^ per well) were added to confluent HUVECs grown on 5-µm pore transwells pre-coated with fibronectin (10 µg/ml). Migrated cells were counted after 3 h in a Casy Counter (Scharfe System).

For time-lapse microscopy, T cells were plated on ICAM-1 or on confluent HUVECs grown on fibronectin-coated coverslips. Images were collected every 30 sec for 1 h on an Axiovert (Zeiss) or a TE2000-E Eclipse (Nikon) inverted microscope with a 20× objective. Cells were tracked with Kinetic Imaging software and analysed with Tempus Meteor software (Andor Technology) and notebooks written in Mathematica 6.0 (Wolfram Research Institute)[49] or with MetaMorph 5.01 software (Molecular Devices).

### Adhesion assay

Starved T cells were labelled with 2′,7′,-bis-(2-carboxyethyl)-5-(and-6)-carboxyfluorescein, acetoxymethyl ester (BCECF; Molecular Probes) in serum-free medium for 20 min, washed with medium containing 1% BSA and incubated with antibodies. Cells (10^5^ per condition) were added to ICAM-1-coated wells for 1 h. Non-adhered cells were washed off and adhesion determined by fluorescence measurement in a Fusion α-FS fluorimeter (PerkinElmer).

### Immunofluorescence

Cells were fixed with 4% paraformaldehyde, permeabilized with 0.2% Triton X-100 in PBS, blocked with 1% BSA in PBS, then stained with antibodies and/or for F-actin using FITC- or TRITC-conjugated phalloidin (Sigma-Aldrich). For pERM staining, cells were fixed with 10% TCA for 15 min at 4°C. Actin in TCA-fixed samples was detected with a goat anti-actin antibody. The following antibodies were used: α-tubulin, ß-tubulin-FITC (Sigma-Aldrich), CD44 (BD Bioscience), ICAM-3 (Abcam), RhoA, actin (Santa Cruz Biotechnology), Rac1, Rac2 (Upstate/Millipore), GFP (Roche), Cdc42, phospho-ERM, and ERM (Cell Signalling). Fluorophore-conjugated secondary antibodies were from Jackson ImmunoResearch. Images were acquired with a 40x/1.3 NA objective using a LSM510 confocal laser-scanning microscope (Zeiss).

### Rho GTPase activity assays

The activity of RhoA, Rac1 and Cdc42 was determined by binding to GST-rhotekin-RBD, GST-PAK-PBD and GST-WASP-PBD respectively [50], [51]. Cells (10^7^ per condition) were harvested in buffer (50 mM Tris HCl pH 7.4, 500 mM NaCl, 10 mM MgCl_2_, 2 mM EDTA pH 8.0, 10% glycerol, 0.5% β-mercaptoethanol, 1% Triton X-100, 0.1% SDS, 0.5% sodium deoxycholate, 1 mM DTT, 0.2 mM sodium orthovanadate, 1 mM PMSF, 1 mM NaF, 1 µg/ml aprotinin and leupeptin); cell lysates were incubated for 45 min with GST-fusion proteins on glutathione-sepharose beads. Bound proteins were solubilized in Laemmli loading buffer and Rho GTPases detected by western blotting. The relative level of active Rho GTPase was quantified by densitometric analysis, normalizing to the ‘total Rho GTPase’ control for each sample.

For active Rac and Rho localization by immunofluorescence, GST-PAK-PBD and GST-Rhotekin-RBD were affinity-purified from bacterial lysates with glutathione-sepharose beads and eluted with 10 mM glutathione in 100 mM Tris-HCl pH 8.0 for 10 min at room temperature. Cells were fixed and permeabilized as described above. After blocking with 3% BSA in PBS for 20 min, the cells were incubated with the eluted GST-PAK-PBD or GST-Rhotekin-RBD (final concentration 2.5 µg/100 µl of blocking solution) for 1 h, followed by rabbit anti-GST antibody (Sigma-Aldrich) and secondary goat anti-rabbit antibody.

### Western blots

Cell lysates were separated in 4–12% Bis-Tris Gels, transferred to Immobilon P membranes (Millipore) and blotted with antibodies to the indicated proteins. The following antibodies were used: anti-RhoA and anti-phospho-stathmin (Santa Cruz Biotechnology), anti-Rac1 (clone 23A8, Upstate), anti-Cdc42, anti-phospho-ERM, anti-ERM and anti-stathmin (Cell Signaling) and anti-GFP (Roche). Secondary antibodies were from Amersham. When required membranes were stripped with 8 M guanidinium hydrochloride (pH 3.0) and re-probed.

### Transfection and live cell GFP visualization

Cells were nucleofected with plasmids using the appropriate Amaxa kit (Amaxa Biosystems) according to the manufacturer's protocol with minor modifications. Briefly, cells were incubated in growing medium without antibiotics for 48 h, then washed and nucleofected (10^6^ cells/100 µl). GFP-Rac1-L61 was a gift from N. Hotchin (University of Birmingham, UK), GFP-Cdc42-L61 from M. Way (London Research Institute, Cancer Research UK), HA-tagged Moesin-TD (T558D) and Moesin-TA (T558A) were from J. Dêlon (Université René Descartes, Paris, France), and EB3-GFP was from A. Akhamanova (Erasmus University, Netherlands).

After transfection cells were cultured for 6 h in growth medium without antibiotics or IL-2. Subsequently cells were incubated in antibody-coated wells as above in starving medium. For migration assays cells were plated on ICAM-1-coated coverslips in starving medium. A series of time-lapse images were collected with a Hamamatsu Orca-ER C4742-95 camera on a Nikon TE2000-E Eclipse Inverted microscope equipped with an environmental chamber, using a 100x/1.4 Plan Apo Ph3 Objective and Andor IQ software (Andor Technology, Belfast, UK). The images were processed with MetaMorph 5.01 software (Universal Imaging Systems, Sunnyvale, CA).

For the visualization of EB3-GFP in static images the 256 greyscale images were converted to indexed color images. Panels to show the track were made by subtracting each frame from the previous frame, producing an image of what had moved between frames. The differences were added together to show all the items moving in the sequence and subsequently converted to indexed color scale.

## Supporting Information

Figure S1 TCR activation impairs T cell polarization but not adhesion. T cells were incubated for 45 min with the indicated antibodies to TCR or control IgG prior to each experiment. (A) T cells incubated with MEM57 and MEM92 antibodies (MEM; kindly provided by Dr. V Horejsi, Institute of Molecular Genetics, Prague, Czech Republic) or control IgG were plated on ICAM-1-coated transwell filters. Migrated cells were counted in the lower chamber after 3 h. Results are shown as percentage of migrated cells, mean of three independent experiments +/− S.E.M. *p<0.05 compared to control, Student's t-test. (B) BCECF-labelled T cells were plated on ICAM-1-coated wells and adhesion determined after 1 h. Results are shown as % of cells adhered to ICAM-1 relative to IgG-pre-treated cells, mean of 3 independent experiments +/− S.E.M. (C) T cells were plated on ICAM-1-coated coverslips. Polarized cells and cells with multiple protrusions (see Figure 2) were counted. The mean of % of cells in 5 experiments +/− S.E.M. is shown (at least 15 cells per experiment). (D) T cells were plated on ICAM-1-coated coverslips and an image acquired every 30 sec. The vector plots show the trajectories of 50 cells from 5 independent experiments with the origin of each cell plotted at the intersection of the axes. (E) T cells were plated on ICAM-1-coated coverslips, then fixed and stained after 1 h with antibodies to the indicated proteins and TRITC-phalloidin to show actin filaments (actin). Confocal images representative of each condition are shown. Scale bars, 20 µm.
(3.35 MB TIF)

Figure S2 Regulation of Rho GTPase activity and localization by TCR activation. (A) GST-PAK-PBD (GST-PBD) staining in cells expressing GFP, GFP-Rac1-L61 or GFP-Cdc42-L61: T cells nucleofected with the indicated plasmids were plated on ICAM-1 and fixed, then incubated with purified GST-PBD followed by anti-GST antibodies, and co-stained for F-actin. Confocal images were acquired with identical settings of contrast and gain. The higher level of staining in cells expressing GFP-Rac1-L61 indicates that GST-PBD recognizes the active form of Rac1. Scale bars, 20 µm (Rac1) or 10 µm (Cdc42). (B) T cells treated with IgG or CD3 for 45 min with or without the Rac inhibitor NSC23766 (1 µM) as indicated were harvested and the activity of Rac1 and RhoA determined in pull-down experiments. Results from a representative experiment of 3 independent experiments are shown.
(1.95 MB TIF)

Movie S1 T cell migrating on ICAM-1 interacting with anti-CD28-coated beads. 1 frame/30 sec for 15 min.
(3.98 MB MOV)

Movie S2 T cell migrating on ICAM-1 interacting with anti-CD3-coated beads. 1 frame/30 sec for 15 min.
(1.90 MB MOV)

Movie S3 IgG-pre-treated T cell migrating on ICAM-1. 1 frame/3 sec for 135 sec.
(3.13 MB MOV)

Movie S4 CD3 pre-treated T cell migrating on ICAM-1. 1 frame/3 sec for 180 sec.
(1.95 MB MOV)

Movie S5 Microtubule dynamics in a control T cell. Cell transfected with EB3-GFP migrating on ICAM-1; 1 frame/5 sec for 150 sec. The left panel shows phase-contrast images, the right panel shows GFP fluorescence.
(0.39 MB MOV)

Movie S6 Microtubule dynamics in a CD3-activated cell. Cell transfected with EB3-GFP and pre-treated with anti-CD3 antibody migrating on ICAM-1; 1 frame/5 sec for 150 sec. The left panel shows phase-contrast images, the right panel shows GFP fluorescence.
(0.77 MB MOV)

Movie S7 T cell expressing GFP-Rac1-L61 migrating on ICAM-1. 1 frame/5 sec for 150 sec. The left panel shows GFP fluorescence, the right panel shows phase-contrast images.
(1.01 MB MOV)
