# Supplementary figures and images for: Rac Activation by the T-Cell Receptor Inhibits T Cell Migration
Source: PLoS One. 2010 Aug 25;5(8):e12393. doi: 10.1371/journal.pone.0012393 (PMC2928276; doi:10.1371/journal.pone.0012393)

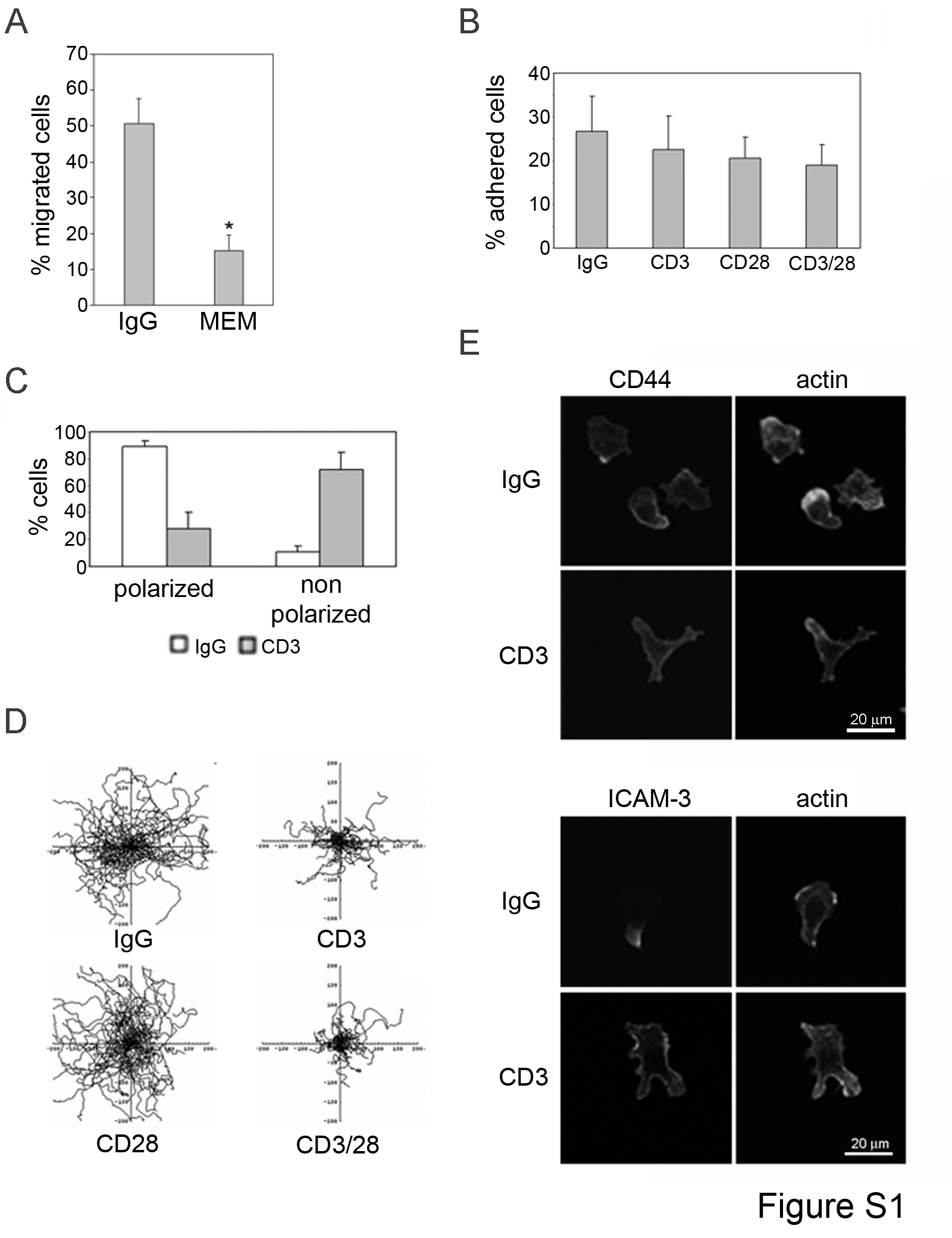

Supplement: Figure S1 — TCR activation impairs T cell polarization but not adhesion. T cells were incubated for 45 min with the indicated antibodies to TCR or control IgG prior to each experiment. (A) T cells incubated with MEM57 and MEM92 antibodies (MEM; kindly provided by Dr. V Horejsi, Institute of Molecular Genetics, Prague, Czech Republic) or control IgG were plated on ICAM-1-coated transwell filters. Migrated cells were counted in the lower chamber after 3 h. Results are shown as percentage of migrated cells, mean of three independent experiments +/− S.E.M. *p<0.05 compared to control, Student's t-test. (B) BCECF-labelled T cells were plated on ICAM-1-coated wells and adhesion determined after 1 h. Results are shown as % of cells adhered to ICAM-1 relative to IgG-pre-treated cells, mean of 3 independent experiments +/− S.E.M. (C) T cells were plated on ICAM-1-coated coverslips. Polarized cells and cells with multiple protrusions (see Figure 2) were counted. The mean of % of cells in 5 experiments +/− S.E.M. is shown (at least 15 cells per experiment). (D) T cells were plated on ICAM-1-coated coverslips and an image acquired every 30 sec. The vector plots show the trajectories of 50 cells from 5 independent experiments with the origin of each cell plotted at the intersection of the axes. (E) T cells were plated on ICAM-1-coated coverslips, then fixed and stained after 1 h with antibodies to the indicated proteins and TRITC-phalloidin to show actin filaments (actin). Confocal images representative of each condition are shown. Scale bars, 20 µm. (3.35 MB TIF) [file pone.0012393.s001.tif]

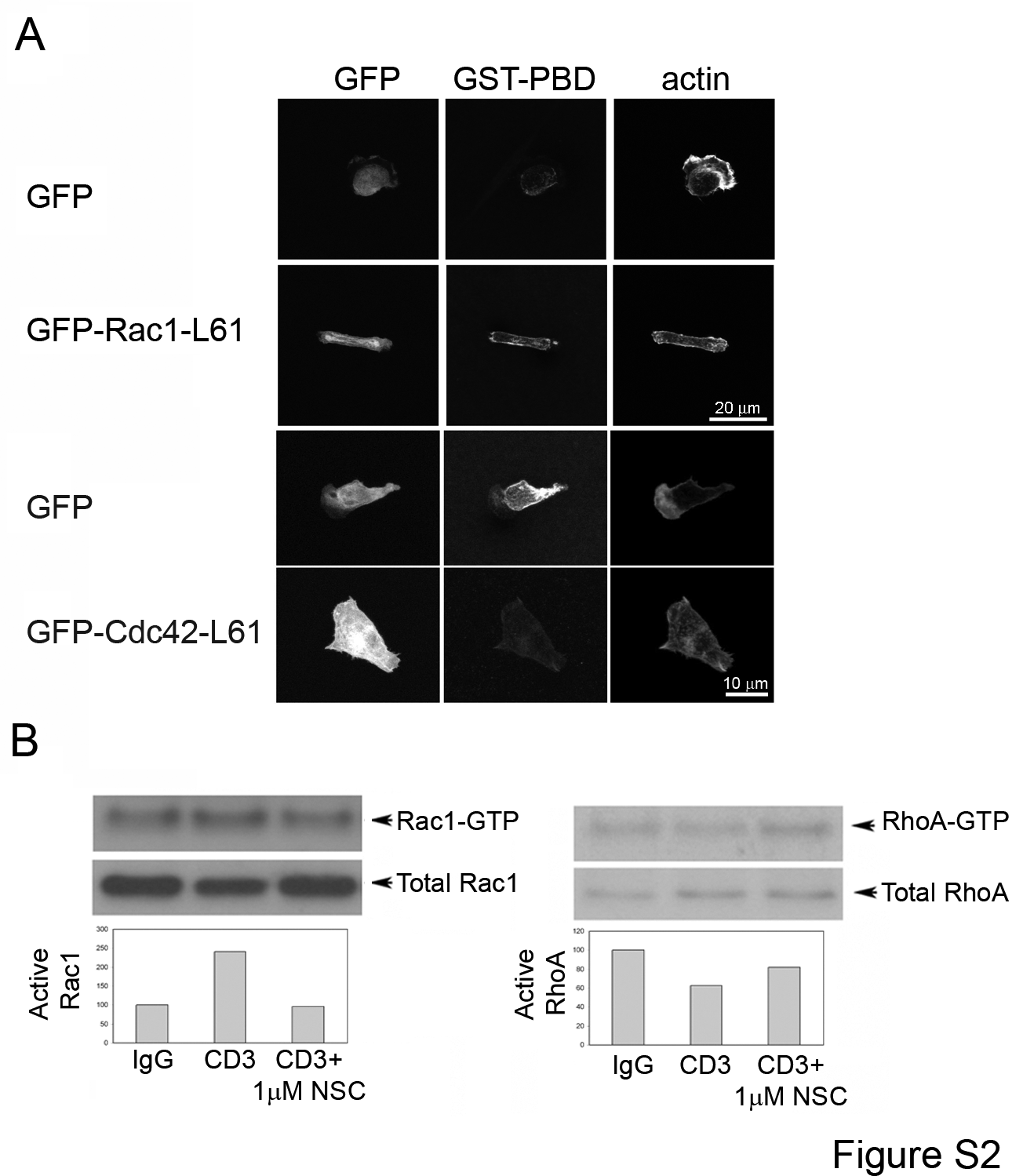

Supplement: Figure S2 — Regulation of Rho GTPase activity and localization by TCR activation. (A) GST-PAK-PBD (GST-PBD) staining in cells expressing GFP, GFP-Rac1-L61 or GFP-Cdc42-L61: T cells nucleofected with the indicated plasmids were plated on ICAM-1 and fixed, then incubated with purified GST-PBD followed by anti-GST antibodies, and co-stained for F-actin. Confocal images were acquired with identical settings of contrast and gain. The higher level of staining in cells expressing GFP-Rac1-L61 indicates that GST-PBD recognizes the active form of Rac1. Scale bars, 20 µm (Rac1) or 10 µm (Cdc42). (B) T cells treated with IgG or CD3 for 45 min with or without the Rac inhibitor NSC23766 (1 µM) as indicated were harvested and the activity of Rac1 and RhoA determined in pull-down experiments. Results from a representative experiment of 3 independent experiments are shown. (1.95 MB TIF) [file pone.0012393.s002.tif]
